# Maternity and Mothering

**Published:** 1911-10-28

**Authors:** 


### MATERNITY AND MOTHERING.

(Inquiries and Correspondence are invited)

### II.—The Care of Children's Teeth.

In a previous article the magnitude of the evils caused and fostered by decayed teeth and the importance both of preventing decay and dealing with it when it occurs were emphasised. Until recent years the prevention of dental caries was difficult or impossible, because the exact methods of its production were unknown or uncertain. All that dentists could do was to repair the ravages already caused, and thus to check in some slight but insufficient degree the further progress of the malady. Now, however, patient research has clearly illuminated every stage of decay of the teeth, and the method of prevention has been completely worked out. Moreover other hypotheses which were once greatly reverenced have been shown not to represent exactly the whole truth, and have been therefore abandoned.

Fortunately the system which expert dental hygienists now propound to the medical profession and the public costs practically nothing; but it requires a certain amount of firmness on the part of parents and guardians, and a certain sense of discipline in the child, and these are two admirable qualities which the twentieth-century mother too seldom succeeds in displaying or evoking.

The system is based upon correct principles of diet. It has been shown that, while the action of bacteria is the fundamental cause of decay of the teeth, that action is only probable when there is prolonged retention or stagnation of fermentable carbohydrates in more or less immediate contact with the teeth, undisturbed by the free access of saliva. When this condition is lacking, the bacteria are, it is true, still present in the mouth; but the normal defensive mechanism of the saliva is then sufficient to arrest their activities and to preserve the teeth against their attacks. It is therefore clear that to avoid any such fermentation of carbohydrate foodstuffs in and about the teeth is to avoid caries, supposing that this theory is correct. The test of actual experiment has shown that this is in fact the case; children brought up very strictly in accordance with these principles have reached adolescence without a single tooth decaying.

It therefore behoves all mothers, and, indeed, all who stand *in loco parentis* to infants or young children, to study very carefully the system of diet, etc., which has produced such results.

First in natural sequence comes the diet of the toothless infant. As is universally conceded this should be the milk of its own mother. Not nearly enough maternal nursing goes on even now, when the disadvantages of substitute feeding have been so thoroughly ventilated and proved.

There are, however, numerous children who are by circumstances denied their natural proper food within a few days or weeks of birth. In institutions where young children are received, such as hospitals, infirmaries, and the like, the proper principles of infant feeding are well understood and practised; so that such infants as a rule suffer to a lesser extent from deprivation of breast-milk than do those who remain in the charge of parents. Among the latter reliance on artificial tinned foods is far too prevalent still, despite the warnings of the medical profession.

The next stage is that of transition from a diet which ought to consist solely of milk to one consisting of a variety of solids, such as is suitable to the digestive system of a child now armed with an efficient apparatus for mastication. In a state of nature, as Dr. Sim Wallace reminds us, it was impossible for mothers to give children food soaked in milk when effecting this transition. Among primitive people this change is effected by giving the growing infant solid substancs to chew at during the intervals between breast feeds. The pap-feeding now in vogue is, according to dental authorities, quite wrong theoretically and practically. The effect of giving an infant, hitherto accustomed to milk, bread well soaked in milk is that the food is gulped down without any retention in the mouth, and consequently without any due admixture with saliva. Not only is ptyalisation of the carbohydrate portion of the food therefore lost, but also the physiological reflex effect on the stomach of proper mastication in the mouth is also wanting.

A little later still, when the temporary molars are erupted, say at something over a year old, the teeth get dirty and tender from want of use; and later still, carious. Thus mastication becomes painful and is more and more neglected by the child; accumulation of carbohydrate refuse around the teeth becomes easier and easier, and the whole of the temporary set may thus become carious, and a septic state of the mouth initiated.

The proper way to start to wean a baby is to give it a piece of some solid food, such as toast, which it cannot swallow without first having thoroughly sucked it. Chewing in the true sense is hardly feasible until the fourteenth month, when the first molars appear; but gnawing and sucking are two processes in which the gums and palate of a baby display a vigour and an efficiency which are truly surprising.

After toast has thus been allowed to supplement a milk diet, say twice a day for a month, rusks and milk-puddings made fairly solid may be allowed; boiled fish and chicken may gradually be added to the dietary in small quantities. The teeth develop well, and when the deciduous set is complete at two to two and a half years of age, the child may, and can, masticate and digest any wholesome food which is a recognised ingredient of a proper adult dietary.

In a concluding article the diet of older children and the other measures necessary to ward off and arrest decay of the teeth will be dealt with. But it should be explained by doctors to all mothers that the care of a child's teeth properly begins at birth; or even beforehand, if we consider the means available to ensure to the prospective mother an adequate supply of breast-milk.

